# Why does Jack, and not Jill, break his crown? Sex disparity in brain tumors

**DOI:** 10.1186/2042-6410-3-3

**Published:** 2012-01-25

**Authors:** Tao Sun, Nicole M Warrington, Joshua B Rubin

**Affiliations:** 1 Department of Pediatrics, Division of Pediatric Hematology-Oncology, Washington University School of Medicine, CB 8208, 660 South Euclid Ave, St Louis, MO 63110, USA

**Keywords:** sex, brain tumors, brain metastases, sexually dimorphic signaling, cytokines

## Abstract

It is often reported that brain tumors occur more frequently in males, and that males suffer a worse outcome from brain tumors than females. If correct, these observations suggest that sex plays a fundamental role in brain tumor biology. The following review of the literature regarding primary and metastatic brain tumors, reveals that brain tumors do occur more frequently in males compared to females regardless of age, tumor histology, or region of the world. Sexually dimorphic mechanisms that might control tumor cell biology, as well as immune and brain microenvironmental responses to cancer, are explored as the basis for this sex disparity. Elucidating the mechanisms by which sex chromosomes and sex hormones impact on brain tumorigenesis and progression will advance our understanding of basic cancer biology and is likely to be essential for optimizing the care of brain tumor patients.

## Review

Primary brain tumors, including both malignant (high-grade) and benign (low-grade) tumors, are highly morbid and life-threatening diseases, particularly as a function of tumor histology, tumor location, and patient age [1-4]. For many common brain tumors, outcome has not improved significantly over the past 25 years despite enormous advancements in neuroimaging, neurosurgery, molecular diagnostics and chemotherapy [5]. Thus, much of current research focuses on those aspects of tumor cell biology and patient characteristics that drive tumorigenesis and resistance to therapy. In this regard, the greater prevalence of primary and metastatic brain tumors in males compared to females, regardless of age, tumor histology and region of the world, strongly suggests that sex is an important determinant of brain tumor biology. Further evaluation of the molecular mechanisms by which sex affects brain tumorigenesis could have significant impact on our understanding of tumorigenesis in the brain and our approaches to treatment of males and females with brain tumors. In the following, we review the epidemiology of parenchymal brain tumors with an emphasis on the relationship between sex and brain tumor incidence, and explore sexually dimorphic mechanisms that could potentially underlie this critical element of brain tumor biology.

### Primary brain tumors occur more commonly in males throughout the world

**Figure 1:**
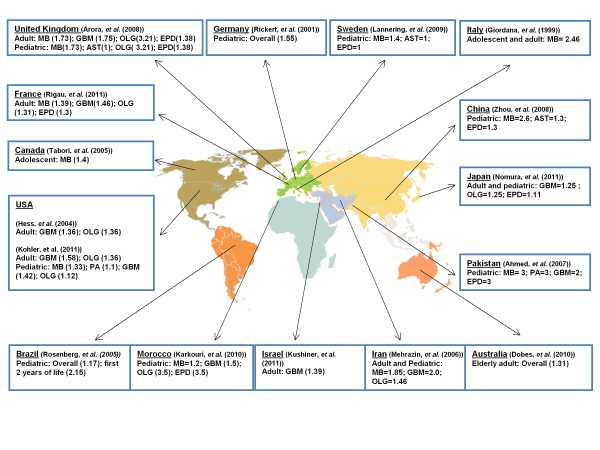
**Sex-based differences in the rates of primary brain tumor exist regardless of age or geographical location**. Reports from around the world describing the incidence of common brain tumors as a function of sex are identified and linked to their countries of origin. Numbers shown in the parentheses are the sex ratio (male/female) of major primary brain tumor types extracted from 16 independent publications from 15 countries, including all major continents and regions of the world (North America, South America, Europe, Middle East, Africa, Asia and Oceania) except for Antarctica. Overall, these studies covered the period of 1974 to 2008, and the sex ratio (male/female) in almost all major brain tumors including both malignant and benign tumors is greater than 1 with a range of 1 to approximately 3.5. See also [2,10-24]. Abbreviations: AST = astrocytoma; EPD = ependymoma; GBM = glioblastoma (multiforme); Glioma, (NOS) = glioma, not otherwise specified; MB = medulloblastoma; OLG = oligodendroglioma; PA = pilocytic astrocytoma.

Cancer rates often exhibit geographical variation suggestive of racial and environmental influences on tumorigenesis [6-9]. To determine whether similar regional and racial factors influence how sex correlates with brain tumor incidence, we retrospectively reviewed 16 independent reports in which data regarding sex and brain tumor histology was readily available [2,10-24]. A clear predominance of brain tumors in males was evident in reports from 15 different countries on 6 continents in which the ratio of cases in males compared to females ranged from > 1 to 3.5 for the major histological subtypes of central nervous system (CNS) parenchymal diseases including astrocytoma (AST), glioblastoma multiforme (GBM), medulloblastoma (MB), ependymoma (EPD) and oligodendroglioma (OLG) (Figure 1). These observations indicate that sex affects brain tumorigenesis through mechanisms that are likely to be independent of race and regional differences in environmental exposures.

In contrast to the above, meningiomas are intracranial tumors that occur more commonly in females than males [25]. However, meningioma is a mesenchymal tumor and not a tumor of the brain parenchyma. Instead, it derives and is generally confined to the meningeal membranes that cover the brain. While the sex disparity in meningioma is fascinating and no doubt important, the extra-axial nature of meningioma separates it from the parenchymal brain tumors described above and it will not be discussed further in this review.

### Primary brain tumors occur more commonly in males regardless of age

For some cancers, the actions of sex hormones can promote or suppress tumor progression. For example, estrogens can promote receptor positive breast cancer development [26] but reduce the risk of gastric cancer [27]. Analysis of brain tumor rates in older men and postmenopausal women, as well as in prepubertal children suggests that sex-dependent differences in circulating sex steroids alone cannot account for the disparity in brain tumor rates in men and women. In Israel and Australia, brain tumors occur more frequently in males older than 65 than in postmenopausal women (M/F ratio = 1.3) [16,18]. In Los Angeles County, California, the overall GBM incidence increased in both sexes between 1974 and 1999. A male predominance in brain tumors was evident throughout this period, and this sex disparity dramatically increased in individuals older than 65 [28].

Brain tumors are the leading cause of cancer-related mortality in children [29]. Childhood brain tumors and adult brain tumors differ in many ways including intracranial locations, histological subtypes, presentation and outcome [30-34]. Even for the same histological subtypes, children and adults can exhibit distinct molecular foundations. For instance, medulloblastoma, the most common malignant brain tumor of childhood, develops infrequently in adults and exhibits age-dependent molecular signatures [35,36]. Similarly, GBM occurs in infants, teenagers and adults with distinct molecular mechanisms and outcomes [37,38]. Despite divergent pathogenesis, male predominance in medulloblastoma and GBM exists in both adult and pediatric populations, even in individuals of less than 2 years [14] (Figure 1). Thus, the influence of sex on brain tumorigenesis is operative regardless of age and sexual maturity, suggesting that it is not a consequence of the acute effects of circulating sex hormones.

### Secondary brain tumors (metastases) occur more commonly in males regardless of the primary cancer type

As many as one-third of all adult cancer cases are complicated by metastases to the brain [39-41]. Brain metastases occur most frequently in lung cancer (48%) and breast cancer (16.5%) followed by a spectrum of other cancers including melanoma, at frequencies less than 10% [40]. A striking disparity in the rates of brain metastases in male and female cancer patients is evident in several large studies involving different primary cancers (Table 1). While rates of primary lung cancer are nearly equivalent in men and women in the US (54% male, (http://www.cdc.gov/cancer/lung/statistics/[42]), between 58% and 83% of all lung-derived brain metastases occur in male patients [43-47]. Similarly, the rates of melanoma are higher among women (http://www.cdc.gov/cancer/dcpc/research/articles/melanoma_supplement.htm, [42]), yet between 58% and 64% of all melanoma-derived brain metastases occur in male patients [48-50]. The sex disparity in brain metastases is evident in tumors derived from different tissues, including those arising from different germ layers such as lung cancer (endoderm and mesodermal origins) and melanoma (neuroectodermal origin), suggesting that it may involve differences in non-cell autonomous effects on cancer cell biology including sexual dimorphism in immune function and the microenvironment of the male and female brains.

**Table 1:** Sex dependent pattern of brain metastases (BM)

Study (year)	Total BM	BM (lung cancer)	BM (melanoma)	Survival
Lagerwaard *et al*. (1999):				
Male	810 (75%)	601 (83%)		
Female	269* (25%)	120 (17%)		
Chao *et al*. (2006):				
Male	687 (61%)			5.8 months
Female	443* (39%)			6.1 months
Videtic *et al*. (2009):				
Male		487 (58%)		5.5 months
Female		348 (42%)		6.3 months
Arrieta *et al*. (2009):				
Male		164 (56%)		
Female		129 (44%)		
Assouline *et al*. (2011):				
Male	164 (78%)			
Female	45* (22%)			
Sampson *et al*. (1998):				
Male			431 (64%)	
Female			239 (36%)	
Hofmann *et al*. (2007):				
Male			77 (58%)	17 weeks
Female			56 (42%)	36 weeks
Raizer *et al*. (2008):				
Male			217 (61%)	
Female			138 (39%)	

*The following number of breast cancer cases was subtracted from the total number of female brain metastasis cases: Lagerwaard (213), Chao (158), Assouline (23).

While the frequency of brain metastases complicating pediatric cancers is far less than in adult cancers, a male-female difference is evident in germ cell tumor (GCT) metastases to the brain with 13/15 reported cases occurring in males [51]. This is particularly interesting given the large disparity in the rates of primary intracranial GCT in young boys and girls. While the reported overall ratio for GCTs (germinoma and non-germinomatous tumors) in boys compared to girls ranges from approximately 3 to as much as 13:1, [52-54], regional variation within the brain is evident. In all, 70% of male GCTs occur in the pineal region and 75% of female GCTs occur in the suprasellar region [55-57]. Within the pineal region male tumors predominate, but in the suprasellar region the ratio of male to female GCTs is more equivalent. The patterns of primary and metastatic CNS GCTs suggest that critical sex and region-dependent interactions occur between GCTs and the brain microenvironment.

### Sexually dimorphic biology may underlie sex disparity in brain tumors

Primary and metastatic brain tumors occur more frequently in males compared to females regardless of tumor type, age, race or region of the world. These observations suggest the existence of sexually dimorphic mechanisms that broadly impact on tumorigenesis and tumor progression. In this section we will examine sexual dimorphism in pathways that could regulate cancer growth and metastasis.

At birth, the brains of human males are 7.8% larger than those of females. The sex disparity in brain size is maintained and increases throughout life, such that the average adult male brain is approximately 11% larger than the female brain [58,59]. These disparities are not a consequence of the acute actions of circulating sex hormones alone, and are instead the result of components of sexual differentiation that are measureable from the time of fertilization. By 2 days post fertilization, male embryos have more cells than female embryos [59,60]. This is well before gonadal differentiation at 8 weeks, and indicates that sex chromosome-based mechanisms of growth regulation exist [60]. Among the genes encoded on the × chromosome that could directly impact growth are glucose-6 phosphate dehydrogenase (G6PD), hypoxanthine phosphoribosyltransferase 1 (HPRT1) and X-linked inhibitor of apoptosis protein (XIAP) [61]. By the blastocyst stage of human development, measureable differences in the expression of these proteins as well as in the autosomally encoded DNA methyltransferases DNMT3 A and B are evident. Thus, at the preimplantation stage of embryogenesis, significant differences in both sex chromosome encoded gene expression and in more global gene expression are observed between male and female embryos [62-65].

In addition to differences in gene expression, male and female embryos exhibit differences in physiology. Total glucose metabolism in human male embryos is twice that of females and male embryos exhibit higher pyruvate and glucose uptake and lactate production than female embryos [62,63,66,67]. Female embryos exhibit greater G6PD expression and activity and four times the pentose phosphate pathway (PPP) activity as observed in males [62,63,66,68]. Alterations in glucose metabolism are required for cancerous growth, and glucose must be shunted from energy production to biosynthetic pathways [69,70]. Cancer cells preferentially employ aerobic glycolysis (the Warburg effect), a non-oxidative form of glucose metabolism characterized by increased glucose utilization and lactate production [71-73]. Whether differences in glucose consumption, lactate production, G6PD and PPP activity in males and females renders them more or less susceptible to oncogenic transformation remains to be determined.

Abnormal activation of the mitogen-activated protein kinase (MAPK) pathway is a common mechanism for dysregulated proliferation and survival in astrocytomas and other cancers [74]. Greater MAPK pathway activation has been observed in male smooth muscle cells *in vitro *[75] as well as in multiple areas of the male brain *in vivo *[76,77]. Estrogens suppress MAPK activity in a sex-dependent manner. *In vitro*, basal levels of activated (phosphorylated) MAPK and extracellular-regulated kinase (ERK) 1/2 were higher in female compared to male astrocytes. However, female astrocytes exhibited greater sensitivity to the MAPK pathway inhibitory effects of estradiol than did male astrocytes [78,79]. Differential sensitivity to the inhibitory effects of MAPK regulation by estrogens was correlated with downstream processes such as proliferation and apoptosis. Furthermore, estrogen treatment increased apoptosis in female cells to a greater degree than in male cells and decreased the percentage of female, but not male, cells in S phase of the cell cycle [78,79]. In the brain, estradiol is produced from testosterone through the actions of aromatase. Aromatase expression is greater in female compared to male astrocytes rendering female astrocytes more sensitive to the effects of testosterone/estradiol than male astrocytes [80]. Interestingly, in an intracranial xenograft model of GBM, estradiol was determined to induce tumor cell apoptosis and promote survival [81].

**Figure 2:**
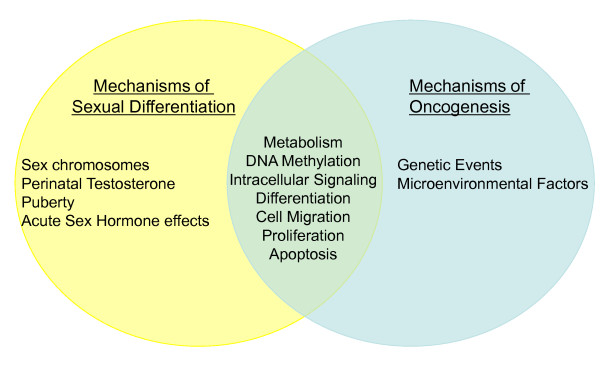
**The mechanisms of sexual differentiation overlap with the mechanisms of oncogenesis**. The process of sexual differentiation shares many fundamental features with oncogenesis, including changes in DNA methylation, glucose metabolism, growth factor signaling, cell migration, proliferation, apoptosis and differentiation.

Among the important targets for estrogen regulation of growth is the cyclic AMP responsive element binding protein (CREB). CREB is a transcription factor that integrates the growth promoting signals downstream of multiple intracellular pathways such as the MAPK, p38MAPK, phosphatidylinositol-3-kinase (PI3K)-AKT and cyclic AMP (cAMP) pathways [82]. Estradiol promotes CREB phosphorylation and interaction with cAMP response elements within the promoter regions of CREB target genes. The action of testosterone-derived estradiol results in higher levels of phospho-CREB in male astrocytes on the day of birth in sexually dimorphic areas of the brain [78]. In addition, the expression of several growth factors including transforming growth factor (TGF) β2 is directly regulated by estradiol in astrocytes [78]. Importantly, TGFβ2 is frequently elevated in cancer cells [83]. Thus, the mechanisms of sexual differentiation share many similarities with the mechanisms of oncogenesis (Figure 2). How the process of sexual differentiation and sexually dimorphic regulation of cellular growth relates to cancer biology should be more fully explored.

### Mechanisms of brain metastases

As first suggested by Paget [84] and demonstrated by Hart and Fidler [85], patterns of solid tumor metastases reflect both tumor cell intrinsic and target tissue microenvironmental factors [86]. In this regard the prevalence of metastases to the brain in male cancer patients is relevant to this discussion.

**Figure 3:**
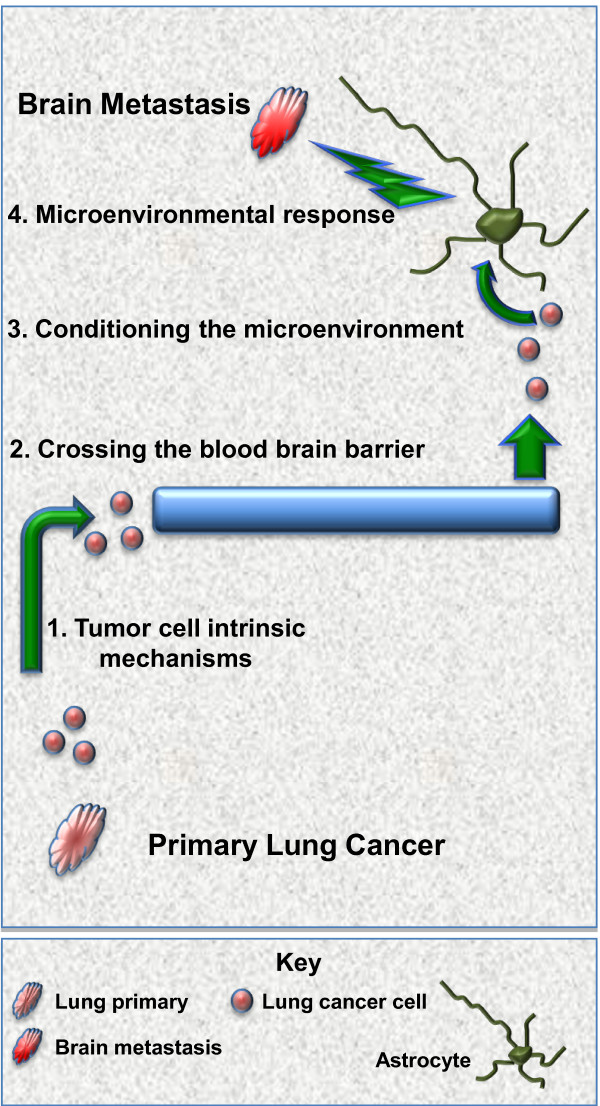
**Potential mechanisms for disparate rates of brain metastases in males and females**. The process of metastasis to the brain involves distinct steps dependent on different genes, pathways and mechanisms. The initial stage (1) involves tumor cell intrinsic mechanisms for the degradation of the extracellular matrix and activation of migratory pathways. This process is often referred to as epithelial to mesenchymal transition (EMT). Pictured is a lung cancer primary tumor and the hematogenous dissemination of lung cancer cells. For metastases to the brain, cells that gain access to the circulatory system and avoid natural killer (NK) cell deletion must cross the blood brain barrier (2). Once present in the brain parenchyma, the lung cancer cells must condition the brain microenvironment for continued growth (3). This process involves secretion of cytokines that induce a microenvironmental response. Pictured is the action of the lung cancer cells on an astrocyte. (4) The activated astrocytes secrete cytokines and growth factors such as interleukin (IL)-6 that promote tumor growth and protect secondary tumors from the cytotoxic effects of chemotherapy.

Current models of metastasis describe a multistage process in which primary tumor cells first gain access to the lymphatics or circulatory system through mechanisms that resemble the normal epithelial to mesenchymal transitions (EMT) that accompany the movement of epithelial cells through the germ layers during early development [87]. An EMT-like phenomenon, including degradation of extracellular matrix and activation of migratory pathways is also observed in cancer, and appears to be essential for metastases [88]. Subsequent establishment of secondary tumors involves avoidance of negative and engagement of positive interactions with the immune system [89], adhesion within target tissues and growth of metastatic lesions through a series of reciprocal interactions between disseminated tumor cells and the microenvironment of target tissues [90,91] (Figure 3).

Each stage of tumor metastasis involves mechanisms that might function in a sexually dimorphic fashion and thereby contribute to the disparate rates of brain metastases observed in multiple cancers. In non-small cell lung cancer, periostin expression, a marker of EMT, was significantly correlated with being male, suggesting that males might possess greater tumor cell intrinsic potential for metastasis [92].

While activation of innate immune cells may promote metastases, it is also likely that prior to the establishment of secondary tumors, tumor cells are vulnerable to immune surveillance and destruction. There are data to suggest that natural killer (NK) cells play an especially important role in this antimetastatic function [93]. NK cell activity displays differential sensitivity to adrenergic suppression in male and female rats, and this increases the susceptibility of male rats to experimental lung metastases. In a lung retention model of intravenously introduced breast carcinoma cells (MADB106), male rats displayed a greater sensitivity to metoproterenol induced β adrenergic activation than female rats [94]. Prior to sexual maturation, differential sensitivity to metoproterenol was not apparent. Further, ovariectomy of female rats had no effect, while castration of male rats abolished the metoproterenol effect in adult animals. These data suggest that sexually dimorphic immune surveillance may impact on tumor metastases in a circulating testosterone-dependent manner.

NK cell function and other components of the peripheral immune response may have limited impact on the behavior of tumor cells once they gain access to the CNS parenchyma. Instead, astrocytes and resident microglia are the predominant components of the CNS cellular immune response. Brain metastases are correlated with significant astrocytic inflammatory reaction in the brain. In response to inflammatory stimuli, astrocytes become 'activated' and in this state secrete a number of cytokines with potential growth promoting properties including interleukin (IL)-1β, IL-3, IL-6, tumor necrosis factor (TNF)α, TGFβ, insulin-like growth factor (IGF)-1 and platelet-derived growth factor (PDGF) [95]. In experiments designed to identify astrocyte-derived factors essential for metastasis growth, IL-1β, IL-6 and TNFα were all found to be capable of stimulating HARA-B lung carcinoma growth [96]. Similarly, coculture of astrocytes with lung or breast cancer cells resulted in the upregulation of survival genes in the cancer cells and the promotion of chemoresistance [97]. Microenvironment-derived interleukin promotion of tumor growth has also been seen with IL-1β stimulation of melanoma [98-100] and IL-6 promotion of primary colon cancer and liver cancers [101,102].

The broad importance of cytokines, especially IL-1 and IL-6 to tumor growth is relevant to sex disparity in brain metastases because astrocyte production of cytokines is sexually dimorphic. While baseline expression of IL-1β, IL-6, interferon-inducible protein (IP)-10 and TNFα was similar in male and female astrocytes, a significant disparity became apparent upon stimulation with lipopolysaccharide (LPS). Under these infection mimetic conditions, expression of IL-1β, IL-6 and TNFα was significantly greater in male versus female astrocytes [103]. Testosterone treatment of neonatal female mice produced a male pattern of cytokine response in adult animals suggesting that sexually dimorphic cytokine production by astrocytes is a stable difference in function that is dependent upon the perinatal surge in testosterone. Similar disparity was observed in liver and colon production of cytokines during primary tumorigenesis of carcinoma [101,102]. In this case, the effects of sex appeared to be mediated by the inhibitory effects of estrogen on IL-6 signaling rather than testosterone dependent sensitivity to immune suppression.

Further evidence for estrogen effects on tumor progression come from the study of sex on tumorigenesis in a mouse model of medulloblastoma genesis as a consequence of mutation in the sonic hedgehog (SHH) receptor patched (PTC). In this model, more than 50% of *Ptc *heterozygous mice develop preneoplastic lesions, but *bona fide *malignancies develop in only 14% to 20% [104,105]. Ovariectomized *Ptc *± mice exhibited a higher rate of tumor progression while estrogen treatment of ovariectomized mice returned the tumor rate to baseline [106]. In this case, the genesis of medulloblastoma is driven by SHH pathway activation. Estrogen did not alter the level of SHH pathway activation. This suggests that estrogen might not exert its effect directly on medulloblastoma cells, but rather might influence microenvironmental function. This interpretation is supported by epidemiological data that suggests SHH-driven medulloblastoma do not occur at different rates in prepubertal boys and girls [107]. Testing this hypothesis would require examining the effects of sex and estrogen on the promotion of SHH-independent tumor types.

Tumor recruitment and activation of astrocytes may not only be important for the formation of metastases, but also in the relative resistance of metastases to therapy. In a model of experimental melanoma metastases, astrocytes protected melanoma cells from the cytotoxic effects of both p-glycoprotein sensitive and insensitive agents [108]. The protection required direct contact between tumor cells and astrocytes, gap junction communication and appeared to be calcium dependent.

Thus, the disparate rates of brain metastases in males and females appear to be a consequence of sexually dimorphic tumor cell intrinsic mechanisms, as well as disparity in immune cell and astrocyte function. Defining the sex-dependent mechanisms that regulate brain metastases could advance efforts to (i) identify patients at the highest risk for metastatic disease, (ii) prevent metastases from occurring, and (iii) improve brain metastasis treatments.

### Sex impacts on outcome from primary and secondary brain tumors

The influence of sex on brain tumors extends beyond tumorigenesis and includes outcome. It has been demonstrated in both pediatric and adolescent groups that males with medulloblastomas have a worse prognosis than females [12,109,110]. Similarly females with either low-grade or high-grade gliomas exhibit higher survival compared to males [111-113]. The survival advantage of being female also extends to brain metastases where women with either lung cancer primaries or melanoma exhibit longer survival compared to men with the same condition [44,46,50].

## Conclusions

The current failure of neuro-oncology to cure more children and adults with brain tumors demands continued effort to understand the molecular basis for brain tumorigenesis and to apply this knowledge to the prevention and treatment of brain tumors. In this regard, the significant impact that sex has on brain tumor incidence and outcome suggests that important oncogenic mechanisms will be revealed through the illumination of how sex affects brain tumorigenesis. It is likely that this will involve cell intrinsic mechanisms regulating metabolism and growth, as well as fundamental mechanisms of cell migration, invasion, immune activity and crosstalk between tumor cells and their microenvironment. At the very least, these efforts should help reduce the rates of brain tumors in males to those in females and improve the outcome for males to equal that of females. Beyond that, one can hope that understanding sex disparity in brain tumors is so fundamental to tumor biology that it will ultimately result in reduced rates of brain tumors and improved outcome for everyone.

## Competing interests

JBR has a patent application pending to use a SNP-based predictor of astrocytoma risk in neurofibromatosis 1 that incorporates the effects of sex.

## Authors' contributions

TS, NMW and JBR each contributed to the literature review and the writing of this manuscript. All authors read and approved the final manuscript.

## Authors' information

JBR is the Co-Director of Pediatric Neuro-Oncology at St Louis Children's Hospital and the Co-Leader of the Cancer and Developmental Biology Program at The Siteman Cancer Center, Washington University School of Medicine. JBR's laboratory research is focused on the mechanisms of brain tumorigenesis in childhood.
